# Investigating social exclusion, affect and emotion regulation in young people using the ostracism online paradigm

**DOI:** 10.1038/s41598-025-09565-z

**Published:** 2025-07-06

**Authors:** Louisa Engelskirchen, Julia Asbrand, Brunna Tuschen-Caffier

**Affiliations:** 1 Department of Clinical Psychology and Psychotherapy, Institute of Psychology, University of Freiburg, Engelbergerstr. 41, 79106 Freiburg, Germany; 2 Department of Clinical Psychology and Psychotherapy for Children and Adolescents, Institute for Psychology, University of Jena, Semmelweisstr. 12, 07743 Jena, Germany

**Keywords:** Adolescence, Ostracism, Affect, Emotion regulation, Acceptance, Internalizing symptoms, Psychology, Human behaviour

## Abstract

Social exclusion significantly affects adolescents’ well-being. Previous research has indicated that factors such as rejection sensitivity, internalizing disorders and dysfunctional emotion regulation can amplify the emotional impact of ostracism, whereas strategies such as acceptance may alleviate it. This study explores affect changes induced by the Ostracism Online paradigm, and examines the roles of rejection sensitivity, emotion regulation and internalizing symptoms in *N* = 73 young people (aged 14–21). The participants were randomly assigned to the experimental and control groups to test whether trained acceptance could support recovery from ostracism. As hypothesized, the results revealed worsened positive and negative affect following ostracism. Other than predicted, rejection sensitivity and internalizing symptoms did not significantly influence these changes. Contrary to expectations, acceptance did not facilitate recovery. The time x group interaction did not reach significance after correction, and post hoc tests did not reveal significant differences. As hypothesized, internalizing symptoms were linked to emotion regulation and rejection sensitivity. These findings contribute to understanding the psychological impact of ostracism, emphasizing the importance of considering mental health in future research, along with the study’s limitations.

**Supplementary Information:**

The online version contains supplementary material available at 10.1038/s41598-025-09565-z.

## Introduction

Social media has become a central aspect of adolescents’ and young adults’ daily lives influencing their social interactions^1,2^. In 2023, 93% of German adolescents used their smartphones for several hours a day, with 88% engaging in social media platforms such as WhatsApp, Instagram, TikTok and YouTube every day^3^. These platforms serve as vital tools for interpersonal communication, for example, through personalized feedback such as likes^4,5^. Alongside the benefits of social connection, social media also presents new avenues for social exclusion^1,6^. Social exclusion, encompassing phenomena like rejection and ostracism, is at focus of experimental research in recent decades^7–9^. Like others^10,11^, we refer to ostracism and social exclusion interchangeably. In addition, research on young people is essential, as this period represents a critical risk phase for the development of psychopathology^12^, while numbers of mental disorders have been increasingly prevalent among youth since years^13^.

Ostracism – being ignored and excluded by others^11^ – threatens basic needs and negatively impacts mood^14,15^. In the context of social media, cyberostracism refers to the experience of being left out of online conversations, group chats, or receiving little interaction^16,17^. A common experience is receiving few or no likes on social media posts, which can evoke feelings of being ignored^5,18^. Research has demonstrated that manipulating social feedback, such as the number of likes, can alter feelings of social inclusion^5^. The Ostracism Online paradigm^5^, an ecologically valid method that mimics a social media platform, has been used to study ostracism and its emotional effects. Various studies utilizing this paradigm have shown that ostracism on social media leads to detrimental effects on emotional and psychological well-being^18–21^. However, most of the work on emotional, cognitive, behavioral, and health-related effects of ostracism is based on adult samples^7^. Research on ostracism, especially on the Ostracism Online paradigm among adolescents is still lacking^22^. This ecologically valid paradigm appears to be highly relevant for adolescent age groups as everyday relevance can be established and it seems to influence affect negatively^10,19^. The Temporal Need-Threat Model suggests that ostracism effects unfold in three stages: the reflexive stage, which involves an immediate, automatic emotional reaction; the reflective stage, where the individual evaluates the situation and considers coping strategies; and the resignation stage, which may occur after prolonged exclusion^23^. Accordingly, affective changes are considered immediate during the reflexive stage.

Social exclusion is considered to be a risk factor for long-term psychological issues, for example, depressive disorders^19,24,25^. Due to intraindividual differences such as psychopathology or rejection sensitivity contributing to feelings of rejection, research on ostracism effects seems to be relevant. Individuals with high rejection sensitivity - a trait characterized by heightened vulnerability to social rejection - tend to experience stronger negative emotional reactions to exclusion^26–29^. This trait has been linked to anxiety and depression, making it a crucial factor in understanding the psychological effects of ostracism^19,27^. Furthermore, rejection sensitivity may exacerbate psychopathological symptoms, particularly in adolescents, where it is assumed to contribute to the development of internalizing disorders^30^. Reinhard et al.^31^ proposed a reciprocal model between ostracism and psychopathology, suggesting that hypervigilance to rejection as well as maladaptive coping mechanisms, both associated with psychiatric disorders, may exacerbate ostracism-related stress, rejection and further psychopathology. Concluding, rejection sensitivity seems to influence ostracism related effects^26,31^, and research on this personality trait in the transition into adulthood is highly relevant^30^. Besides, only a few studies exist linking the influence of rejection sensitivity and worsening affect after ostracism^26,28^.

Another influencing factor being associated with rejection sensitivity and internalizing symptoms are emotion regulation (ER) deficits^30^. ER is defined as the process by which people decide what emotions they have, when they have them and how they express and experience them^32^. Deficits in ER are considered as a transdiagnostic factor in psychopathology^33,34^. They can be conceptualized as general difficulties with ER as well as dysfunctional strategy use (i.e., more maladaptive and fewer adaptive strategies), and are associated with internalizing symptoms^33,35^. In the context of social exclusion, ER can be referred to as improving emotional states elicited by exclusion situations^36^. While first reactions to ostracism, such as changes in mood, occur at the reflexive stage, coping and regulation strategies intervene in the transition to the reflective stage according to the Temporal Need-Threat Model^16,23^. Even though there seems to be a natural decrease in ostracism effects, it remains unclear how individual differences contribute to recovery^22^. ER strategies could contribute to making the exclusion situation seem less threatening and support recovery from the stressful situation^16,36^. Nevertheless, there is overall a lack of empirical findings investigating regulation possibilities for social exclusion^16^. Theoretical work suggests different strategies for coping with social exclusion. Riva^36^ discussed cognitive and behavioral avoidance and approach strategies for regulation during the reflective stage. Timeo et al.^16^ proposed psychological strategies to help young people cope with the negative effects of social exclusion. They differ between strategies for restoring basic needs and changing perspective. While strategies intended to restore basic needs seem to be helpful after Ostracism Online in a preadolescent sample^20^, findings on changing perspective strategies are sparse, especially in youth samples. Both theoretical approaches propose acceptance as a possibly functional strategy. While it is considered a functional cognitive approach strategy in one framework^36^, it is, in another, conceptualized as part of mindfulness - a strategy aimed at changing one’s perspective^16^. The latter includes attention and acceptance of the situation and upcoming thoughts without judgement. It was previously trained in adult samples, with promising results concerning reducing negative emotions and supporting recovery after rejection^16,37^. However, it has not yet been studied in a youth sample.

Building upon this theoretical framework, and combining it with ER research, we focus on acceptance as a stand-alone ER strategy. Acceptance is defined as being aware of thoughts and emotions without judging or willing to change them^38,39^ and is classified as a putatively adaptive strategy^35^. While the results of studies on training acceptance in laboratory settings are mixed^39–42^, the literature in general speaks in favor of the functionality of acceptance^33^. Studies with adult samples indicate no superiority of acceptance over cognitive reappraisal regarding effects on positive (PA) and negative affect (NA) after negative mood induction^39,43^. Interestingly, acceptance is assumed to be easier to learn and implement than cognitive reappraisal, as it requires fewer cognitive resources and is perceived as less demanding to apply^39,43^. Previous laboratory studies training acceptance in young people focused on inducing specific emotions instead of a general NA or PA and had no or differing definitions of acceptance^41,42^. While Volkaert et al.^41^ and Wante et al.^42^ reported promising short-term effects of acceptance on both NA and PA states in youth, it is important to note that the effects of acceptance were tested in comparison to other regulation strategies, some of which outperformed acceptance in their respective study designs. Regarding the short-term effectiveness of acceptance in regulating affective states, research remains inconsistent - particularly among youth populations - highlighting the need for further investigation. According to the strategy-situation fit hypothesis^44^ ER is thought to depend on contextual factors, such as the controllability of the situation. Given that ostracism typically occurs without explanation or overt negative attention^11^, acceptance may be particularly useful in coping with this type of uncontrollable social stressor^45^. In the context of psychopathology, especially internalizing disorders such as anxiety and depression, reduced use of acceptance has been identified as a transdiagnostic factor^33,35^.

The present study focuses on changes in affect after being ostracized, how those changes may be regulated with an acceptance instruction and how individual differences influence affect in an adolescent to young adult sample. This study is among the first to explore whether acceptance can function as an adaptive stand-alone strategy in this specific context, using *Ostracism Online* as a negative mood induction in a controlled laboratory setting. This research seems especially relevant for the intended age group. In most studies, the influence of psychopathological symptoms was not taken into account. As the mean peak age for any mental disorder is 14.5 years, and nearly half of all mental disorder have a peak onset before the age of 18, it seems highly relevant^12^. Especially when considering the interrelations between internalizing disorders and ostracism effects^31^, rejection sensitivity^27^ and ER difficulties^30,35^. We consider internalizing symptoms (depression and anxiety disorders) transdiagnostic and dimensional, as this approach allows variation in symptom severity and comorbidity^46^. Since we employ a dimensional approach, participants with and without diagnosed mental disorders within the internalizing spectrum will be included.

Concluding, we investigated if the Ostracism Online paradigm leads to reduced self-reported PA and greater NA compared to baseline measurement. Considering individual differences, we examined if participants with higher rejection sensitivity would show a greater increase in NA and whether those with higher internalizing symptoms would exhibit both a greater increase in NA and decrease in PA. Based on studies training ER strategies in youth samples^41,42^, we investigated if using acceptance as an ER strategy after negative mood induction by Ostracism Online would lead to an increase in PA and a decrease in NA compared to the control group. Finally, we examined if participants with higher self-reported internalizing symptoms habitually use more maladaptive and less adaptive ER strategies, experience greater ER difficulties, and report higher rejection sensitivity.

## Method

This study was part of a larger project about ER strategy training in laboratory and everyday life (for project description see https://osf.io/mr7w5). Approval for the study was obtained from a local ethics committee. All study procedures were carried out in accordance with the Declaration of Helsinki. The study was preregistered at OSF (https://osf.io/fd8u3).

### Participants

Adolescents aged 14–21 years were recruited via flyers, outpatient clinics, student WhatsApp groups, participant databases and civil registers. Participants were reimbursed with vouchers up to 50€ for local stores upon completing the entire project. Inclusion criteria required signed informed consent (including legal guardian consent for minors), and fluency in German. Participants with confirmed anxiety disorders (separation anxiety disorder, specific phobia, social anxiety disorder, agoraphobia, generalized anxiety disorder, panic disorder) and/or depressive disorders (major depression, dysthymia), as well those with subclinical symptoms or no symptoms, were included. Exclusion criteria were pervasive developmental or neurological disorders, acute suicidality, acute psychosis and current alcohol or substance dependence. There were no restrictions based on demographic characteristics.

The targeted sample size for the overall project was *N* = 100 evenly distributed between experimental (*n* = 50) and control (*n* = 50) group. An a priori power analysis using G*Power version 3.1.9.7^47^, indicated that with an expected 10% exclusion rate, the study had 80% power to detect a small effect size (*d* = 0.3) in t-tests. Of the 99 participants who completed the study, *n* = 26 failed manipulation checks and were excluded, leaving a final sample of *N* = 73 (*M* = 17.4, *SD* = 2.5). Consistent with similar studies females were overrepresented (86.3% female, 9.6% male, 4.1% diverse)^10^. All the participants reported owning a smartphone, with 78.1% being active on social media. See Table 1 for sociodemographic characteristics.

**Table 1:** Sociodemographic characteristics by group.

	Experimental group (*n* = 36)		Control group (*n* = 37)	
	*M*	*SD*	*M*	*SD*
Age	17.1	2.5	17.7	2.6
	*n*	%	*n*	*%*
Gender				
Female	32	88.9	31	83.8
Male	4	11.1	3	8.1
Diverse	0	0	3	8.1
Highest education				
None	15	41.7	12	32.4
Middle school	0	0	2	5.4
Secondary school	8	22.2	7	18.9
High school	12	33.3	16	43.2
Bachelor degree	1	2.78	0	0
Current occupation				
Education	1	2.8	1	2.7
Studies	10	27.8	16	43.2
Work	2	5.6	0	0
School	23	63.9	20	54.1
History of psychotherapy^a^	13	36.1	14	37.8
Current	5	38.5	6	42.9
Past	8	61.5	8	57.1
Experience with ER^a^	16	44.4	20	54.0
Social media^a, b^	26	72.2	31	83.8

^a^ answered with yes. ^b^ active on social media.

* ER*  emotion regulation.

### Measures

#### Affect

State affect was assessed using a translated version of the Positive and Negative Affect Schedule for Children (PANAS-C)^48^. The German equivalents of the original English items were sourced from Großheinrich^49^ and compared with the long version by Laurent et al.^50^. Participants rated their current PA (“joyful”, “cheerful”, “happy”, “lively”, “proud”) and NA (“miserable”, “mad”, “afraid”, “scared”, “sad”) on a 5-point Likert scale from 1 (*very slightly or not at all*) to 5 (*extremely*). Both the 5-item PA and NA scale met the cutoff for good internal consistency (Cronbach’s alpha > 0.80) in the original English version^48^, with our sample showing good internal consistency (PA: α = 0.89, NA α = 0.84).

#### Rejection sensitivity

Rejection sensitivity was assessed using either Part I of the Questionnaire for Assessing Rejection Sensitivity in Children and Adolescents (German: Fragebogen zur Zurückweisungsempfindlichkeit bei Kindern und Jugendlichen, FZE-K)^51,52^ or the shortened 9 item version of the Rejection Sensitivity Questionnaire (RSQ)^53,54^, depending on participants’ age to ensure relevance to their life experiences. The FZE-K, used for underaged participants, includes nine social interaction scenarios where rejection is possible, assessing both anxiety and the expectation of rejection. Responses are rated on a 6-point scale. The FZE-K demonstrated good internal consistencies ranging from *α* = 0.78 to *α* = 0.82^51^. In our sample the internal consistency for the items of the weighted score of the FZE-K was good with α = 0.81. Anxiety (α = 0.78) and expectation (α = 0.76; see supplementary material for additional information) were acceptable.

For participants aged 18 and older, the RSQ-9 was administered. Like the FZE-K, it assesses rejection sensitivity by asking participants to imagine themselves in various social situations and rate their anxiety and expected rejection on a 6-point scale. Participants were asked to imagine what it would be like if they were personally in the described situation and to rate how they would feel. The German version of the RSQ has good psychometric properties^54^. For the short version the reliability was good (0.80)^53^. Internal consistencies in our overaged sample for the RSQ-9 were α = 0.86 for all items, α = 0.79 for anxiety items and α = 0.79 for expectation items.

#### Psychopathology

Trait-level psychopathological symptoms were measured using the Brief Symptom Checklist (BSCL)^55^, which assesses 53 physical and psychological symptoms over the past seven days on a 5-point Likert scale (1 = *not at all*; 5 = *extremely*) The BSCL includes psychological stress subscales and three global indicators. For this study only the scales relevant to internalizing symptoms (anxiety, phobic anxiety, depression) were analyzed. Internal consistencies (Cronbach’s alpha) for these scales are acceptable (α = 0.72; phobic anxiety), to good (α = 0.80; anxiety) and very good (α = 0.88; depression)^55^.The internal consistencies in this sample were lower with α = 0.65 (phobic anxiety), α = 0.72 (anxiety), and α = 0.78 (depression). Combined, the three scales, labelled internalizing symptoms in the following, showed good internal consistency with α = 0.87.

#### Emotion regulation

Trait ER strategies were assessed using the Questionnaire for Assessing Emotion Regulation in Children and Adolescents aged 10–20 years (German: Fragebogen zur Erhebung der Emotionsregulation bei Kindern und Jugendlichen; FEEL-KJ)^56^. The questionnaire measures 15 ER strategies across three emotions – fear, sadness, and anger – with three items each on a 5-point scale (*almost never* to *almost always*). For practical purposes, only fear and sadness were assessed. The questionnaire captures both adaptive strategies (e.g., problem-solving, distraction, acceptance) and maladaptive strategies (e.g., giving up, aggression, withdrawal), along with unclassified strategies (e.g., social support). Internal consistencies (Cronbach’s alpha) for adaptive strategies were excellent (α = 0.93) and good for maladaptive strategies (α = 0.82). In the present sample internal consistencies are comparable with α = 0.92 for adaptive (see supplementary material for further information) and α = 0.89 for maladaptive strategies.

ER difficulties were measured using the German version of the Difficulties in Emotion Regulation Scale (DERS)^57,58^. The 36 item scale in the original version^59^ assesses six subscales of emotion dysregulation (non-acceptance of emotional reactions, problems with goal-directed behavior, impulse control problems, lack of emotional attention, limited access to ER strategies, and lack of emotional clarity) using a 5-point scale indicating how often the items are applied (1 = *almost never*,* 0–10%*, to 5 = *almost always*,* 91–100%*). Higher values indicate greater difficulties in ER. Internal consistencies in the overall scale were good (α = 0.93, ranging from α = 0.80 to α = 0.89 in the subscales)^59^. The German version assessed in adolescents aged 13–20 years had similar internal consistencies (α = 0.95)^58^, comparable to the present sample (α = 0.94).

### Ostracism online

For inducing negative mood through social exclusion the Ostracism Online paradigm (see http://smpo.github.io/socialmedia/ for a demo version and details) by Wolf et al.^5^ was used, translated to German and adapted for the age group. Participants were told that they would interact with peers in an online task, but they were the only real person involved; other group members were preprogrammed profiles. Separate versions of the task were used for ages 14–17 and 18–21, differing only in profile descriptions for better relating with the age and interests of the group members. Participants entered initials, a nickname, or a pseudonym and chose one of 50 avatars (as in the original study taken from https://pickaface.net/), representing diverse appearances. They wrote a short introduction text. Afterwards, the participants were introduced to nine other profiles, could read and like them for 3 min, and were instructed to form realistic impressions. They were asked to read the profiles carefully as they may be questioned about them later. The introduction page, resembling a social media page, displayed 10 profiles, including their own, with a 3-minute timer. Each profile showed a counter of received likes, and ostracism was manipulated by the participant receiving only one like. The success of the manipulation was assessed by using the translated manipulation check based on Wolf and colleagues^5^.

### Acceptance instruction

Participants received standardized instructions to apply acceptance as an ER strategy. Specifically, they were instructed to recall a personally experienced situation from the past seven days that had elicited negative or unpleasant emotions. While reflecting on this event, they were asked to observe and allow any arising thoughts and feelings without attempting to suppress, change, or avoid them. The instruction was adapted from an Acceptance and Commitment Therapy exercise intended for accepting anxiety^60^. As we were interested in the whole spectrum of negative or unpleasant emotions, we changed the instructions respective. The wording was adapted for youth participants and the context. The translated protocol is provided in the supplementary material.

### Manipulation checks

We adapted and translated the manipulation check assessing direct and indirect assessment of participants’ level of engagement, as described in the original Ostracism Online paradigm study^5^, for checking attentiveness during the paradigm and if technical difficulties occurred. The translated items and answer options are presented in the supplementary material. Furthermore, we used the state version of the Emotion Regulation Questionnaire (ERQ State-Short)^61^ to look at ER strategies used after the ostracism paradigm, similar to other studies^45^. To date, there is no validated questionnaire that assesses situational ER strategies. The ERQ State-Short assesses the strategies reappraisal, avoidance, rumination, acceptance, and distraction with three items each on a scale from 1 = *strongly disagree* to 5 = *strongly agree*. Internal consistencies (Cronbach’s alpha) in our sample were mixed (acceptance: α = 0.51, reappraisal: α = 0.53, rumination: α = 0.68, distraction: α = 0.74, avoidance: α = 0.82).

### Procedure

**Fig. 1:**
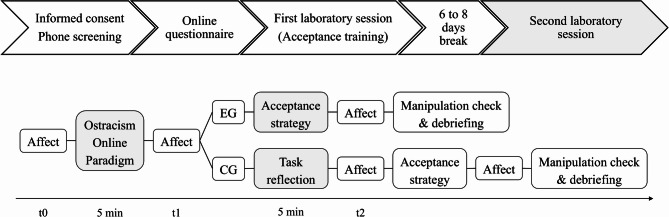
Study procedure (upper panel) and structure of the second laboratory session (lower panel).

See Fig. 1 for a visualization of the study procedure. After signed informed consent was obtained, exclusion criteria were assessed through a phone screening. Participants completed an online baseline questionnaire via formr^62^, which collected demographic information (age, gender, education level, current occupation, history of therapy, prior experience with ER, smartphone ownership and social media use), as well as data on psychopathological symptoms, ER and rejection sensitivity.

As the project was divided into two experimental sub-studies, each comprising an experimental and control group, participants were randomly assigned to one of the four possible combinations (two groups per sub-study) using adaptive randomization with an equal allocation ratio (1:1:1:1). Since psychopathology was assessed using a dimensional approach, participants were not categorized into clinical or nonclinical groups. The randomization sequence was generated using the randomizeR package (version 2.0.0) in R (version 4.0.5)^63^. Following randomization, participants attended an initial laboratory session in which they received the acceptance instruction. At the end of the session, participants were informed that they would apply this strategy during their next session by themselves. The second laboratory session took place six to eight days later, whenever feasible. As a baseline measure, participants rated their current affect, then performed the Ostracism Online task and rated their current affect afterwards. Participants in the experimental group were instructed to apply the acceptance strategy they had learned. Those in the control group, in a waiting condition, were asked to reflect on the profiles without any specific ER instructions (e.g., Which profile did you particularly like? Who do you think you would get al.ong with in real life?). This supported the cover story, which informed them that they should pay close attention to the profiles as they might be asked questions about them later. See supplementary material for translated instructions. After three minutes, all participants rated their affect once again. Following this, the control group also received the acceptance instruction and provided a third affect rating. Both groups completed manipulation checks and rated the credibility of the profiles and the task itself.

### Data analytic strategy

The analyses were performed using R version 4.4.1^64^. Welch two sample t-tests were used to test for group differences in descriptive statistics. Reliability measures were obtained using the psych R package^65^. Following Wolf et al.^5^ participants were excluded due to failed manipulation such as technical difficulties, multitasking, or lack of attention to both their own and others’ “likes”. For the FEEL-KJ we computed mean scores for the anxious and sad ER scales. After adapting coding for FZE-K and RSQ as provided by each questionnaire and looking at differences between scores, we treated measures as one scale.

After checking the assumptions for each statistical test, dependent t-tests were used to analyze affect changes before and after Ostracism Online, and Cohen’s d was computed as effect size. Simple regression analyses were employed to assess whether changes in affect were predicted by rejection sensitivity and internalizing symptoms. Due to criticisms of composite scores like for the FZE-K and RSQ, additional analyses were conducted using separate scales (anxiety/concern about the outcome, expectation of acceptance/rejection), as well as their interaction^66^. These results are presented in the supplementary material. Furthermore, 2 × 2 mixed ANOVAs with repeated measures were used to analyze pre- and post acceptance NA and PA between experimental an control group. Although t-tests were initially proposed in the preregistration, ANOVAs were determined to be more appropriate for hypothesis testing. Finally, regression analyses were performed to examine the effects of internalizing symptoms on ER strategies (adaptive and maladaptive), ER difficulties and rejection sensitivity, and changes in NA and PA. For all statistical tests an alpha level of 0.05 was set. Bonferroni-Holm correction was applied to control for multiple comparisons. In case of assumption violations regarding homoscedasticity and normality of residuals in regression analyses, HC4 estimators were used to calculate robust standard errors^67,68^. Given that ANOVAs are generally robust to normality assumptions^69^ and assumption violation for the homogeneity of variance primarily affects unequal group sizes^70^, we proceeded without corrections when detected. Due to the dimensional approach, outliers deemed plausible.

## Results

Participants were excluded when dropping out before completing the second laboratory assessment (*n* = 6), lacked sufficient German language proficiency (*n* = 1), or failed manipulation check (*n* = 26) in accordance with Wolf et al.^5^. No participant reported technical difficulties, and most remained on the web page and read most of the profiles (90.4% reported reading all). Exclusions were primarily due to inattention to their own “likes” (*n* = 22) or “likes” others received (*n* = 17). Participant flow is detailed in Figure S1 of the supplementary material. Descriptive statistics per group and correlations of main variables are provided in Table 2. There were no significant differences on relevant variables (*p* > .05) between groups.

**Table 2:** Descriptive statistics by group and correlation coefficients of main variables at baseline.

Variable	EG (*n* = 36)	CG (*n* = 37 )						
	*M* (*SD*)	*M* (*SD*)	1	2	3	4	5	6
1. Internalizing symptoms	10.2 (8.8)	10.6 (7.5)						
2. ER difficulties	86.4 (25.6)	91.5 (22.5)	0.68					
3. Adaptive ERS	44.5 (9.0)	43.6 (9.4)	− 0.38	− 0.54				
4. Maladaptive ERS	27.4 (7.9)	29.0 (5.3)	0.71	0.78	− 0.34			
5. Rejection sensitivity	10.9 (4.9)	10.5 (3.9)	0.46	0.58	− 0.49	0.53		
6. Negative affect (t0)	1.4 (0.5)	1.6 (0.7)	0.55	0.44	− 0.05	0.31	0.28	
7. Positive affect (t0)	2.8 (0.9)	2.8 (0.9)	− 0.39	− 0.39	0.40	− 0.43	− 0.40	− 0.13

* EG* experimental group, *CG *control group,* ERS * emotion regulation strategies,* ER * emotion regulation.

**Fig. 2:**
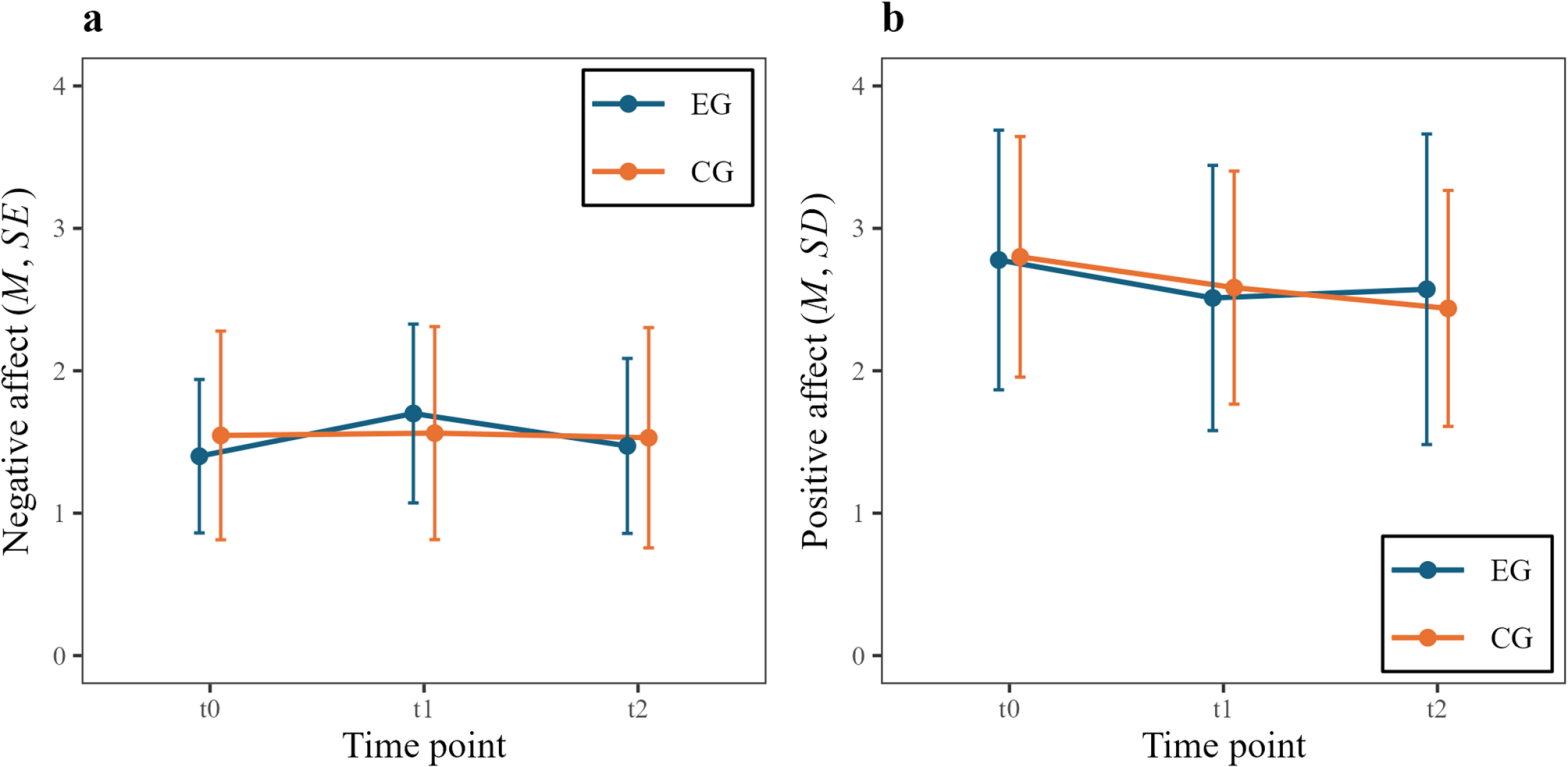
Negative (**a**) and positive (**b**) affect in experimental and control group over all time points.* EG*  experimental group,* CG* control group, t*0 * baseline, *t1 * after Ostracism Online, *t2 * after acceptance vs. waiting condition.

The overall sample was predominantly healthy concerning internalizing symptoms (*M* = 10.4, *SD* = 8.1, range 0–42, with a possible range of 0–68). Since underage and overage participants did not differ significantly in rejection sensitivity scores (*t*(64.9) = -1.07, *p* = .291), data from the FZE-K and RSQ-9 were combined for analysis. Overall, participants reported rather low scores of rejection sensitivity, with scores ranging from 3.4 to 26.2 (*M* = 10.7, *SD* = 4.4), well below the possible maximum of 36. For visualization of PA and NA across the measured time points in both groups, see Fig. 2.

Given the predominantly female sample and the wide age range during adolescence, we conducted additional analyses controlling for age and gender. Neither variable had a significant influence on the results; thus, we proceeded without accounting for age or gender. As outliers were detected, sensitivity analyses excluding outliers were performed. As they produced similar results, we proceeded with the analyses without excluding them.

### Worse affect after ostracism online paradigm

We conducted paired, two-sided t-tests to assess differences in affect before and after the paradigm across groups. NA was significantly higher after the paradigm (*M*_Diff_ = -0.16, *t*(72) = -2.99, *p* = .003, *d* = 0.35, 95% CI [-0.26, -0.05]) and PA was significantly lower after the paradigm (*M*_Diff_ = 0.24, *t*(72) = 3.87, *p* < .001, *d* = 0.45, 95% CI [0.11, 0.37]). Both results remained significant after correction for multiple testing (NA: *p*_adj_ = 0.023, PA: *p*_adj_ = 0.002).

Given that two versions of profiles were used for underaged and overaged participants, we checked for differences in reported affect between the age groups. No group differences were found for NA (*t* = -0.07, *p* = .943) or PA (*t* = 0.95, *p* = .347).

### Rejection sensitivity and internalizing symptoms on affect

Following Reich et al.^10^ we computed the difference in NA and PA score (post minus pre-manipulation) to examine affect change. Mean scores were *M* = 0.2 (*SD* = 0.5) for NA and *M* = -0.2 (*SD* = 0.5) for PA in the entire sample. The weighted rejection sensitivity score did not significantly predict changes in NA (*F*(1, 71) = 1.72, *p* = .264, *R*^*2*^ = 0.02). Internalizing symptoms did not significantly predict changes in NA (*F*(1, 71) = -1.41, *p* = .474, *R*^*2*^ = 0.01) or PA (*F*(1, 71) = -0.07, *p* = .943, *R*^*2*^ = -0.01).

### Acceptance and affect changes

Statistically significant interactions between group and time were observed for NA (*F*(1, 71) = 4.52, *p* = .037, η_p_^*2*^ = 0.06) and PA (*F*(1, 71) = 4.91, *p* = .030, η_p_^*2*^ = 0.07). After correction for multiple testing, the interactions missed significance (NA: *p*_adj_ = 0.150, PA: *p*_adj_ = 0.150 ). Pairwise comparisons revealed no significant differences between the experimental and control group or between time points. See Table S1 and Table S2 in supplementary materials for full ANOVA results.

No group differences emerged between reported state acceptance during the task (experimental group: *M* = 10.7, *SD* = 2.2; control group: *M* = 11.3, *SD* = 2.0). Regarding other strategies assessed with the ERQ State-Short, no significant group differences were found (*p* > .05). In the post assessment participants reported trying to apply acceptance during the task (*M* = 72.1, *SD* = 20.79 on a 0-100 scale). Furthermore, 83.6% of participants agreed that they were well-prepared for using acceptance in this situation, and 74.0% felt confident that they were able to apply what they had learned in the training session.

### Internalizing symptoms

**Fig. 3:**
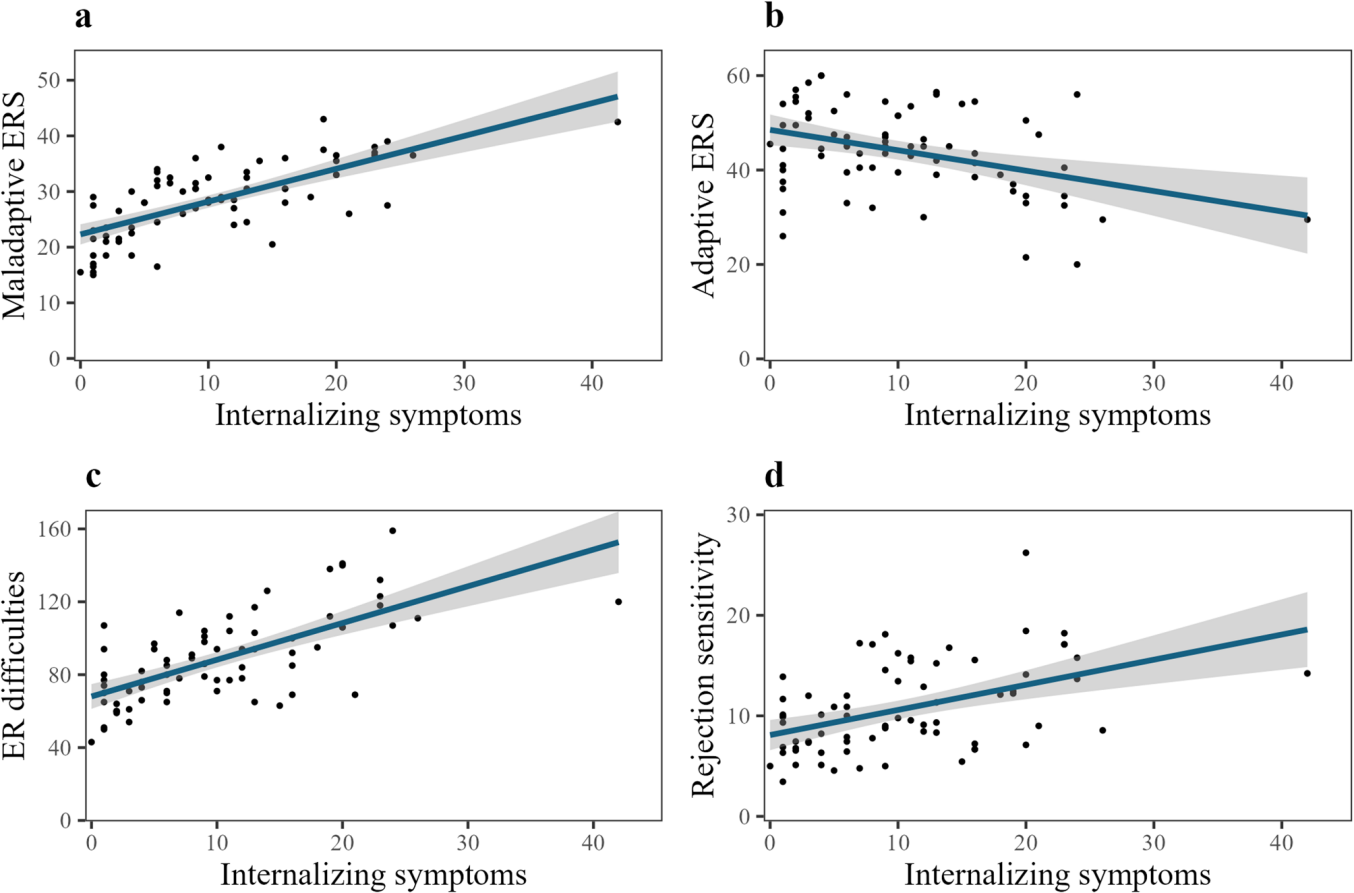
Visualization of linear regression models. Relationship of internalizing symptoms with maladaptive strategies (**a**), adaptive strategies (**b**), emotion regulation difficulties (**c**), rejection sensitivity (**d**).* ERS* emotion regulation strategies, *ER * emotion regulation.

Participants reporting more internalizing symptoms were found to use more maladaptive ER strategies (*F*(1, 71) = 72.64, *p* < .001, *R*^*2*^ = 0.50), fewer adaptive strategies (*F*(1, 71) = 12.12, *p* < .001, *R*^*2*^ = 0.15), and experience greater difficulties with ER (*F*(1, 71) = 60.27, *p* < .001, *R*^*2*^ = 0.46). They exhibited higher rejection sensitivity (*F*(1, 71) = 18.99, *p* < .001, *R*^*2*^ = 0.21). After adjusting for multiple testing, all analyses remained significant (maladaptive *p*_adj_ < .001, adaptive *p*_adj_ = .006, difficulties *p*_adj_ < .001, rejection sensitivity *p*_adj_ < .001). See Fig. 3 for a visualization of the linear regression models.

### Exploratory analyses

Given the significant reduction in PA following Ostracism Online, we analyzed whether rejection sensitivity influenced the change in PA, similar to the analysis of NA in the second hypothesis. The weighted rejection sensitivity score did not predict the change score in PA *F*(1, 71) = 2.17, *p* = .145, *R*^*2*^ = 0.03.

To consider further individual differences we investigated whether ER difficulties and strategies predict change scores from first to second time point (post-manipulation). In the experimental group subsample (*n* = 36), we tested whether trait variables (symptoms, ER difficulties and strategies) predicted change scores from the second to the third time point (post-acceptance). Only the use of adaptive strategies significantly predicted changes in positive affect in the experimental group, *F*(1, 34) = 2.86, *p* = .007, *R*^*2*^ = 0.17.

## Discussion

This study aimed to evaluate the suitability of the Ostracism Online paradigm for inducing affect changes in adolescents in a controlled laboratory setting and to examine the influence of rejection sensitivity and internalizing symptoms on affect changes. Additionally, we investigated whether pre-trained acceptance supports recovery from ostracism effects. Finally, the study explored the role of dimensional internalizing symptoms in ER and rejection sensitivity.

Consistent with our expectation, we observed affective changes after Ostracism Online. Adolescents reported increased NA and decreased PA following the paradigm, with small to medium effect sizes for NA and medium effects for PA. These findings align with prior studies on adolescents^19^ and adults^10^, showing that exclusion from significant others worsens affect^14,15^. In line with assumptions of the Need Threat Model, effects occurred as affective changes immediately after ostracism experience^23^. Thus, the Ostracism Online paradigm^5^ successfully induced affective changes in this study, emphasizing the relevance of social exclusion by significant others in online contexts, such as social media^1,6^, and expanding results from online studies to a laboratory setting^19^. However, interpretation should consider limitations, as we did not directly assess whether participants felt rejected. Affect changes may therefore stem from factors beyond the paradigm, although most participants reported finding the task and profiles credible during debriefing. Since social media platforms are an integral part of the reality of many young people’s lives^1,2^ and social exclusion is considered as a risk factor as well as maintaining factor among others for psychological disorders^25,31^, further research is warranted. Especially for exploring long-term effects, as most studies on ostracism effects are cross-sectional^71^.

Contrary to our prediction, individual differences such as rejection sensitivity and internalizing symptoms did not contribute to stronger affective reactions after Ostracism Online. While previous research suggests that rejection sensitivity is associated with increased negative reactions in exclusionary situations^26–29^, participants in our study with heightened rejection sensitivity did not show increased NA in change scores. Given that both NA and PA worsened according to the first hypothesis, we conducted an exploratory analysis on the relationship between rejection sensitivity and PA change. Similar to NA, no significant association was found. One possible explanation is the low scores of rejection sensitivity in our sample, potentially resulting in less vulnerability to exclusion situations in the sample. Assuming reasons other than the feeling of rejection for affective changes, rejection sensitivity would not have been relevant. In the definition of social exclusion, rejection and ostracism are treated as different constructs^8^. Nevertheless, both experiences have similar negative consequences, such as the feeling of being ignored^8^, which is not directly assessed with the rejection sensitivity questionnaires used. Regarding the interplay with psychopathological symptoms, for example, in the suggested reciprocal model^31^, rejection sensitivity may not be interpreted independently. Furthermore, internalizing symptoms did not predict increased NA or decreased PA. Contrary to assumptions^31^, participants with more internalizing symptoms did not exhibit stronger emotional reactions to Ostracism Online. Importantly, internalizing symptoms were assessed dimensionally and not categorially which poses challenges for comparability. Furthermore, despite efforts to recruit participants across the symptom spectrum, most had low internalizing scores, which may not have been sufficient to elicit stronger affective responses. Moderate to strong baseline correlations were observed between internalizing symptoms and NA (positive association), as well as with PA (negative association), suggesting that these affective states were already present prior to the ostracism manipulation and were thus unlikely to be substantially influenced by it. Nevertheless, further research is needed to investigate individual differences in the context of ostracism paradigms in youth populations.

The hypothesis that acceptance as an ER strategy improves affect has to be rejected after correction for multiple testing. The observed group × time interaction effects, although with medium effect sizes, did not remain significant after correction. This aligns with studies showing no effects of acceptance in regulating elicited emotions^72^, or supporting results of acceptance being less effective in affect improvement than other strategies^39,41^, and meta-analyses indicating no superiority of acceptance in reducing NA^73^. A former study investigating coping strategies after Ostracism Online in a youth sample found strategies to be helpful concerning restoring basic needs^20^ rather than affect. While the assumption that psychological strategies potentially support recovery from ostracism effects^16^ seems theoretically promising, further investigations for statistical support are warranted. Participants in the experimental group did not report greater use of acceptance than the control group, and no group differences emerged for other strategies. Post-debriefing feedback revealed some difficulties in recalling and applying the acceptance instructions, raising questions about the effectiveness of the manipulation. While acceptance, as defined in the present paper, has theoretical implications for uncontrollable situations, such as ostracism, those assumptions lack statistical findings. Possible explanations include high variances in reported affective states and a smaller sample size, as actually needed for detecting medium effects in a mixed ANOVA. Additionally, more arousing paradigms or a focus on specific emotions^41,42^ may yield stronger effects. Acceptance may have impacted stress or physiological responses, like skin conductance level^39^, which were not measured. The acceptance training was designed for this study and was not tested for efficacy. Additionally, it may not have been of sufficient duration or intensity to demonstrate significant effects. Although the intervention was intended to target acceptance as an ER strategy, the instructional content also included elements such as observation and self-compassion, which are typically associated with broader mindfulness-based approaches. This is particularly important in light of recent evidence indicating that school-based mindfulness interventions often show limited efficacy in adolescents, especially when compared to active control conditions^74–77^. This may limit conclusions about the specific effects of acceptance, as the influence of other mindfulness components cannot be ruled out. Notably, both groups received the acceptance training early in the study, and the control condition may have unintentionally encouraged regulation. While the control group was instructed in thinking about the other profiles, the experimental group received explicit acceptance instructions. The control group instruction may have also lead to some type of regulation. Descriptive trends suggest a natural decline in ostracism effects over time^22^, independent of condition. As high variances emerged, it can be assumed that the use of acceptance might have benefited some participants. However, exploratory analyses revealed no impact of internalizing symptoms or regulation difficulties on affective change in the experimental group, individuals who generally used more adaptive strategies tended to have increased positive affect following acceptance. Further research should focus on more diverse youth samples and a broader symptom spectrum to better understand individual differences in the effectiveness of acceptance-based regulation.

According to our final hypothesis, dimensional internalizing symptoms were associated with different relevant variables as expected. Participants with more internalizing symptoms used more maladaptive and fewer adaptive ER strategies, had greater ER difficulties and reported higher rejection sensitivity. These findings align with previous research linking psychological disorders, such as depression and anxiety, to dysfunctional ER^33,35^. Effect sizes were medium to large for adaptive strategies, and large for maladaptive strategies and ER difficulties. We also found a medium to large association between internalizing symptoms and rejection sensitivity, extending existing literature^19,27^. However, our findings are not generalizable to adolescents with diagnosed depressive and anxiety disorders, as internalizing symptoms were assessed dimensionally and the sample was predominantly healthy. Nevertheless, the results underscore the importance of considering psychopathological symptoms in adolescents when researching relevant related constructs. In cases where clinical group differences are not of main interest, a dimensional approach might be suitable. Furthermore, intercorrelational associations suggest an interplay between constructs such as psychopathology, rejection sensitivity and emotion dysregulation^31^, which should be further examined in future studies.

While this study contributes to ostracism research, several limitations should be considered. First, we did not directly assess whether participants felt excluded, so we cannot confirm the effectiveness of the paradigm^10^. Additionally, instructions provided before the baseline questionnaire may have induced stress, potentially affecting baseline measurements. Variability in experimenters due to scheduling could have also introduced inconsistencies in baseline data. The 6- to 8-day gap between lab sessions may have been too long for some participants to retain the acceptance training, as some reported difficulties in recalling the instructions. Engaging in practice during the days following the training may have supported consolidation and reduced the likelihood of forgetting the acceptance instructions. Nevertheless, the majority of participants reported feeling well-prepared and confident in their ability to apply what they had learned. Furthermore, some scales used, like FEEL-KJ and ERQ State-Short, have not been fully validated for the whole age group or have known reliability issues when self-assessed, particularly among individuals with low emotional awareness^78^. We also combined the FZE-K and RSQ scales to ensure age-appropriate situations, but this decision should be considered in interpretation. The reliance on self-report measures may have led to biases, suggesting a need for additional objective physiological or observational data in future research. Higher participant exclusion rates compared to similar studies^5,10,19^ were mostly due to failed attention, which may have impacted the credibility of the paradigm in a laboratory setting. This, combined with the smaller sample size as calculated, may have contributed to some nonsignificant results. Moreover, nearly 50% of participants reported that the profiles seemed dissimilar to themselves, although this feedback was given after they learned that the profiles were fake, which could have influenced their responses. A total of 22% reported no active engagement on social media, potentially limiting the relevance of the Ostracism Online paradigm for this group, as they lack familiarity with social media dynamics and the experience of receiving or not receiving social feedback, such as likes. Consequently, future studies should account for and control social media use where applicable. Finally, while lab-based studies offer control, using the Online Ostracism paradigm in a more realistic online setting might better capture the nuances of online social interactions and improve external validity.

Despite its limitations, this study has notable strengths. Our findings contribute to ostracism research in young people, especially regarding the Ostracism Online paradigm in a laboratory setting. We successfully recruited a quite diverse group of young people, concerning education and current occupation, encompassing middle adolescence to emerging adulthood. As no group differences in demographic data were found, the randomization was successful. Using a dimensional approach to assess psychological symptoms allows for a more nuanced understanding of participants’ experiences, as it is more sensitive to variations in symptom severity than categorical measures^79^. While the sample was predominantly healthy, there was still variability in internalizing symptom severity, with 37% reporting experiences with therapy (and *n* = 11 being currently in therapy). Additionally, the acceptance training was based on recent personal experiences of the participants, making the task emotionally relevant and engaging. Although we did not analyze the specific events, the recency criterion (within the last 7 days) likely ensured their salience, enhancing the ecological validity. Finally, manipulation checks were conducted to confirm the effectiveness of the paradigm.

Future studies should integrate objective measures such as heart rate variability or electrodermal activity to complement self-report data, particularly for assessing immediate effects of ostracism paradigms^80^. This could help mitigate social desirability bias and provide a more comprehensive understanding of emotional regulation and stress responses. The incorporation of eye-tracking technology could provide insights into participants’ attentional focus during tasks, improving the validity of manipulation checks. To enhance participant focus and reduce the cognitive load, future research could provide instructions in audio format or a combination of both^39^, allowing for better task engagement. It would be beneficial to explore dimensional measures with a wider range of symptom severity. Regarding control groups, including an over-inclusion and neutral control group, for example, with a filler-task, alongside the experimental exclusion group, could help assess the paradigm’s effects more effectively^19,81^. Given the limitations of laboratory settings, conducting the paradigm in an online context should be considered, despite authors suggesting its applicability in offline settings^5^. Results from laboratory studies may not be generalizable to real-life experiences of social exclusion^9,82^. To address this, ecological momentary assessment studies would be suitable^82^. Additionally, modifying the cover story, such as having participants posting status updates^10^, could enhance realism. Future research should also investigate the interactions among relevant variables, as the correlations found suggest potential intercorrelations among constructs that warrant further exploration. Concerning the acceptance training, future studies should consider the interval between sessions, the intensity of training and outcome measures apart from affective responses^39^.

Taken together, this study is among the first to employ the Ostracism Online paradigm in a laboratory setting with adolescents, examining the role of acceptance as a strategy for regulating the effects of ostracism. Our findings highlight the need for further exploration of the interrelations among relevant variables, real-life ostracism experiences, and effective strategies for facilitating recovery from ostracism effects. Such insights are crucial for supporting adolescents in social exclusion situations.

## Electronic supplementary material

Below is the link to the electronic supplementary material.

Supplementary Material 1

## Data Availability

The datasets analyzed during the current study are not publicly available due to reasons of privacy but are available from the corresponding author upon reasonable request.
