# Supplementary material for: Investigating social exclusion, affect and emotion regulation in young people using the ostracism online paradigm
Source: Sci Rep. 2025 Jul 6;15:24110. doi: 10.1038/s41598-025-09565-z (PMC12230144; doi:10.1038/s41598-025-09565-z)
Supplement: Supplementary file 1 — Supplementary Material 1 [file 41598_2025_9565_MOESM1_ESM.docx]

Supplementary materials:

**Investigating social exclusion, affect and emotion regulation in young people using the Ostracism Online Paradigm**

Louisa Engelskirchen^1^*, Julia Asbrand^2^, and Brunna Tuschen-Caffier^1^

^1^ Department of Clinical Psychology and Psychotherapy, Institute of Psychology, University of Freiburg, Engelbergerstr. 41, 79106 Freiburg, Germany

^2^ Department of Clinical Psychology and Psychotherapy for Children and Adolescents, Institute for Psychology, University of Jena, Semmelweisstr. 12, 07743 Jena

* [louisa.engelskirchen@psychologie.uni-freiburg.de](mailto:louisa.engelskirchen@psychologie.uni-freiburg.de)

**Supplementary methods**

**Cronbach’s alpha for adaptive emotion regulation strategies**

Item 15 from strategy forgetting in the anxiety scale showed a negative correlation with other items. As the analysis including the adaptive scale when excluding forgetting strategy didn’t change results, we proceeded as suggested by the authors with item 15 included.

**Cronbach’s alpha for FZE-K**

The first item of the expectancy scale had negative correlations after item was recoded. As results from analyses didn’t change when excluding the first item, we proceeded with item 1 included as authors suggested.

**Translated Protocol of Acceptance Instruction**

Make yourself comfortable in your chair, with the soles of your feet resting firmly on the floor and your back sitting upright. Your arms and legs are uncrossed, and your hands are resting gently on your thighs. You can keep your eyes closed or open them and let your gaze drop. Let your gaze become soft and relaxed.

Take some time now and focus on the sensations in your body. Pay special attention to areas where you feel touch or pressure. Where does your body come into contact with the chair? How does the seat feel? Is it warm or cold, hard or soft? Can you feel your feet resting on the floor? Take your time and settle in slowly. There’s nothing else you need to do right now except sit here.

Bring your attention to your breath. Take a deep breath into your belly and slowly exhale through your nose. Feel how your belly rises as you inhale and sinks back down as you exhale. Observe the natural flow of your breath—how the cool air enters your body and leaves it warmer. Try not to control your breathing rhythm; just let it flow naturally.

I’d now like to invite you to place yourself as fully as possible into the situation you thought of earlier. You don’t need to give any answers—just reflect quietly in your mind.

- Where were you at that moment?
- How did the situation come about?
- Who was involved?

When you feel ready, direct your attention to the thoughts and emotions you had in that situation.

- What thoughts were going through your mind at the time?
- What kinds of feelings came up?

Turn toward the thoughts and emotions that arise as you remember the situation. Try to become aware of what’s happening inside you as you think about it. It’s perfectly natural for your thoughts to drift - to worries, images, bodily sensations, or emotions. Notice these thoughts and feelings and stay with them.

Allow even your uncomfortable feelings to be there. What unpleasant emotions come up? Where in your body do you feel them? Don’t push them away - stay with them. Whether it’s doubt, worry, fear, nervousness, anger, or sadness - let them be. Accept that they are here in this moment. Give them space by allowing them to be present.

How does it feel for you to simply let everything come up without pushing it away? Do you feel the need to fight it or make it stop? Or can you simply let it be there and consciously say: It’s okay that you’re here. I feel you, and I allow this experience. I let you be and I accept you.

If the sensations or discomfort grow stronger as you recall the situation, acknowledge this and stay with it. Breathe along with your discomfort and accept the feelings. See if you can give this feeling a place in your body. Maybe you can create space for it in your heart and meet it with kindness and compassion.

Allow all sensations and thoughts that arise while remembering the situation. Your mind may step in with judgments and tell you that it’s getting worse. If that happens, thank your mind for its input—it’s only trying to help.

Then gently return to your present sensations as they truly are, not as your mind suggests. Let your thoughts remain just thoughts, your feelings just feelings. Give your sensations as much attention as they need. And when you notice that your uncomfortable feelings no longer demand your attention, gently let them go.

This exercise is now coming to an end. Slowly become aware of the space around you. Take one more deep breath in and out. If you like, you can loosen up your body by shaking out your arms and legs or stretching. Then open your eyes or lift your gaze and let it become clear. Take your time to fully arrive back in the here and now.

**Translated Instructions after Ostracism Online**

Experimental Group: “Lean back and take a deep breath in and out. If you’d like, you can close your eyes. Tune in to yourself: What feelings and thoughts are present right now? Try to become aware of what’s going on inside you at this moment. Allow even your uncomfortable feelings to be there. Accept that they are here right now. Whatever emotions come up, let them be without judging them. You can consciously say to them: It’s okay that you’re here. Repeat the exercise until the time runs out.“

Control Group: “You now have three minutes to reflect on the following questions. Use the entire time for this exercise, even if it feels long. What do you think about the other group members? How likable do you find them? Which profile did you particularly like? Who do you think you would get along with in real life? Which profile did you find less likable? You have time to reflect until the time runs out.”

**Translated Manipulation Check Items based on Wolf et al. (2015)**

| **Item** | **Answer options** |
| --- | --- |
| Did you spend the entire 3 minutes on the task? | Yes, the whole time  Most of the time  **No, I did other things** |
| Did you read the other participants’ texts? | Yes, all of them  Yes, most of them  Some  **No, I didn’t read any of the texts.** |
| Did you pay attention to how many “likes” you received? | Yes  **No** |
| Did you pay attention to how many “likes” others received? | Yes  **No** |
| Do you think the other young people in the group are similar to you? | No, not at all  Somewhat dissimilar  Somewhat similar  Yes, very similar |
| How easy was it for you to imagine the others in real life? | Not easy at all  A little  Very easy |

*Note*. Bold-marked answer options were defined as exclusion criteria.

**Supplementary results**

**Figure S1**. Flowchart of Participants.

Enrollment

Assessed for eligibility (*n* = 106)

Excluded (*n* = 1)

- Declined to participate (*n* = 1)

Randomized (*n* = 104)

Included in study (*n* = 49)

Analyzed (*n* = 36)

Included in study (*n* = 50)

Allocated to experimental group (*n* = 53)

- Received allocated intervention (*n* = 50)
- Dropouts (*n* = 3)

Allocated to control group (*n* = 51)

- Received allocated intervention (*n* = 49)
- Dropouts (n = 2)

Analyzed (*n* = 37)

Allocation

Baseline

Analysis

Excluded (*n* = 14)

- Failed manipulation check (*n* = 14)

Excluded (*n* = 12)

- Failed manipulation check (*n* = 12)

Excluded (*n* = 1)

- Did not meet inclusion criteria (*n* = 1)

**Additional calculations for rejection sensitivity based on Meule (2022)**

According to Meule (2022), the sensitivity scales anxiety and expectation and their interaction were analyzed separately. Neither the anxiety scale (*F*(1, 71) = 0.68, *p* =.474, *R^2^* = 0.00), the expectation scale (*F*(1, 71) = 1.37, *p* =.286, *R^2^* = 0.02), nor their interaction (*F*(3, 69) = 0.48, *p* =.162, *R^2^* = 0.01) significantly predicted the change score in negative affect. Neither did the anxiety scale (*F*(1, 71) = 3.92, *p* = .052, *R^2^* = 0.05), expectation scale (*F*(1, 71) = 0.52, *p* =.474, *R^2^* = 0.01), nor their interaction (*F*(3, 69) = 1.51, *p* =.221, *R^2^* = 0.06) predict change score in positive affect.

As for the influence of internalizing symptoms on rejection sensitivity, we examined the influence of internalizing symptoms on the anxiety scale (*F*(1, 71) = 16.19, *p* <.001, *R^2^* = 0.19), expectation scale (*F*(1, 71) = 7.26, *p* =.009, *R^2^* = 0.09), and their interaction (*F*(1, 71) = 16.87, *p* <.001, *R^2^* = 0.19).

**ANOVA tables for acceptance and affect changes**

**Table S1.** Means, Standard Deviations, and Mixed Analyses of Variance in Negative Affect.

| Effect | df | MSE | *F* | η_p_*^2^* | *p* |
| --- | --- | --- | --- | --- | --- |
| Group | 1,71 | 0.89 | 0.07 | .00 | .798 |
| Time | 1,71 | 0.08 | 8.02 | .10 | .006** |
| Group x time | 1,71 | 0.08 | 4.52 | .06 | .037* |

*Note. ** p* < .01, * *p* < .05.

**Table S2.** Means, Standard Deviations, and Mixed Analyses of Variance in Positive Affect.

| Effect | df | MSE | *F* | η_p_*^2^* | *p* |
| --- | --- | --- | --- | --- | --- |
| Group | 1,71 | 1.62 | 0.02 | .00 | .884 |
| Time | 1,71 | 0.08 | 0.82 | .01 | .367 |
| Group x time | 1,71 | 0.08 | 4.91 | .07 | .030* |

*Note.* * *p* < .05.
